# Uniaxial‐Oriented Perovskite Films with Controllable Orientation

**DOI:** 10.1002/advs.202401184

**Published:** 2024-03-11

**Authors:** Dongni Li, Xiangyu Sun, Yao Zhang, Zhen Guan, Yansong Yue, Qingya Wang, Lu Zhao, Fangze Liu, Jing Wei, Hongbo Li

**Affiliations:** ^1^ Beijing Key Laboratory of Construction‐Tailorable Advanced Functional Materials and Green Applications Experimental Center of Advanced Materials School of Materials Science and Engineering Beijing Institute of Technology Beijing 100081 China

**Keywords:** film properties, orientation, perovskite, phase separation, stability

## Abstract

Perovskite films with large crystal size, preferred orientation, and facile fabrication process, combining advantages of single‐crystal and polycrystalline films, have gained considerable attention recently. However, there is little research on the facet properties of perovskite films. Here, (111)‐ and (001)‐oriented perovskite films with bandgaps ranging from 1.53 to 1.77 eV, and systematically investigated their orientation‐dependent properties are achieved. The (111)‐oriented films show electron‐dominated traps and the (001)‐oriented films show hole‐dominated traps, which are related to their atomic arrangement at the surface. Compared with the (001)‐oriented films, the (111)‐oriented films exhibit lower work function and superior water/oxygen robustness. For the wide‐bandgap films, the lattice of the (001)‐oriented film provides an unobstructed passage for ion migration. Comparably, the (111)‐oriented films exhibit suppressed ion migration and excellent phase stability. The optimized unencapsulated solar cells based on both (001) and (111) orientations show a similar high efficiency of ≈23%. The (111)‐oriented solar cell exhibits excellent stability, maintaining 95% of its initial efficiency after 1500 h maximum power point (MPP) tracking test, and 97% initial efficiency after 3000 h aging in ambient conditions. This work paves the way for the rational design, controllable synthesis, and targeted optimization of uniaxial‐oriented perovskite films for various electronic applications.

## Graphical abstract

The (111)‐ and (001)‐oriented perovskite films with bandgaps ranging from 1.53 to 1.77 eV, and systematically investigated their orientation‐dependent properties, including defect density, carrier mobility, surface work function, environmental stability, and ion migration are achieved. This work paves the way for the rational design, controllable synthesis, and targeted optimization of perovskite films for various electronic applications.

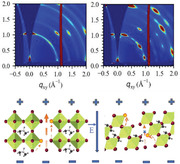

## 1 Introduction

Metal halide perovskites are expected to lead the revolution in photovoltaics with their unique optoelectronic properties of strong light absorption, long carrier diffusion length, superb defect tolerance, and tunable bandgap.^[^ ^1^, ^2^, ^3^, ^4^ ^]^ Up to now, the power conversion efficiency (PCE) of metal–halide perovskite solar cells (PSCs) has surpassed 26%.^[^ ^5^, ^6^ ^]^ However, the further commercialization of PSCs is hindered by their short lifetime which is ≈1 order of magnitude lower than silicon solar cells.^[^ ^7^ ^]^

The development of semiconductor optoelectronics has largely relied on the use of single crystals for their nearly perfect crystal structure, uniform electrical properties, high chemical purity, and facet‐dependent performance.^[^ ^8^, ^9^, ^10^, ^11^, ^12^, ^13^, ^14^, ^15^, ^16^, ^17^ ^]^ Unfortunately, challenges for single‐crystal perovskite photovoltaics remain in terms of material growth, uncontrollable thickness, and area,^[^ ^18^, ^19^, ^20^, ^21^, ^22^ ^]^ unsatisfactory contact between single‐crystal and charge transport layers^[^ ^23^, ^24^ ^],^ and high surface defect density.^[^ ^25^ ^]^ As a result, the maximum PCEs of single‐crystal‐PSCs are still far below their polycrystalline counterparts.^[^ ^12^ ^]^ It is worth noting that the polycrystalline films prepared by solution‐process are still greatly attractive due to their facile, controllable, and low‐cost fabrication process,^[^ ^26^, ^27^, ^28^ ^]^ although the existence of grain boundaries and surfaces may lead to massive trap densities and reduce the PCE and stability of PSCs.^[^ ^29^, ^30^ ^]^

Combining the advantages of single and polycrystalline perovskites, perovskite films with large crystal sizes, preferred orientation, and facile fabrication processes have attracted a lot of attention.^[^ ^31^, ^32^, ^33^ ^]^ On one hand, compared with polycrystalline films, the higher phase purity and larger grains of perovskite films could effectively reduce the grain boundary and thus suppress defects and ion migration^[^ ^34^, ^35^ ^]^; on the other hand, the single orientation of perovskite provide an effective model for further investigating the working principles of PSCs that are related to the surface and grain boundaries.^[^ ^36^ ^]^ For example, the (220) facet of MAPbI_3_ single crystal exhibits higher electron and hole conductivities, and the (112) facet shows enhanced ionic conductance than that of (100) facet.^[^ ^37^, ^38^ ^]^ The density functional theory calculations have revealed that the (110), (111), and (211) facets could introduce additional trap states, while the (100) facet is free from trap states.^[^ ^39^, ^40^, ^41^ ^]^ Similarly, the FAPbI_3_ grains also show facet‐dependent stability where the (111) facet is much more resistant to moisture than the (100) facet.^[^ ^14^ ^]^ These results indicate that exquisite control of film orientation is an effective strategy to further optimize the photovoltaic performance of PSCs.

Although previous research has demonstrated the anisotropic properties of perovskites, such as photoelectric properties, carrier mobility, and defect distribution, and emphasized the impact of orientation on device performance, they mainly focus on single‐crystal perovskites or different grains in polycrystalline films. Few studies have focused on the orientation‐dependent properties of perovskite films. Ma et al.^[^ ^42^ ^]^ reported that carrier mobility and photocurrent of the (100) and (111) facets of FAPbI_3_‐based perovskite film are much higher than those of the (110) facets. However, Zhang et al.^[^ ^43^ ^]^ stated the opposite conclusion that the (110) facet had higher conductivity, enhanced charge carrier mobility, and better‐matched energy level alignment compared to the (100) facet. Therefore, systematic investigations about the controlled synthesis and anisotropic film properties of perovskite films with different orientations are urgently needed to achieve PSCs with higher PCE and enhanced stability.

In this work, we synthesized Cs_0.03_(FA_0.90_MA_0.10_)_0.97_Pb(I_1‐x_Br_x_)_3_ (x = 0.1–0.4) perovskite films with (111)‐ and (001)‐preferred orientations by antisolvent engineering. All of the films were deposited with the same process, providing an objective platform to evaluate the intrinsic orientation‐dependent film properties. For both normal bandgap (NBG) and wide bandgap (WBG) perovskite films, the (111)‐oriented films exhibit lower hole trap density and higher electron trap density than the (001)‐oriented films, which are related to their surface termination and defects types on different crystal facets. Moreover, the (111)‐oriented films exhibit better stability than (001)‐oriented ones due to the inorganic PbI_6_ octahedra layer on their surface. For WBG perovskite, the commonly used (001)‐oriented films show severe phase separation as the ion migration channels are parallel to the direction of the built‐in electric field. In contrast, the (111)–oriented films exhibit excellent phase stability as it block ion migration channels along the direction of the electric field. Solar cells based on both (111) and (001)‐oriented films show the champion PCEs of ≈23% for NBG PSCs and ≈19.8% for WBG PSCs. The (111)‐oriented PSCs exhibited better stability as it maintained 97% initial PCEs after 3000 h aging in ambient conditions. This work provides guidance for controllable synthesis and targeted optimization of perovskite films with different orientations, as well as rational design for novel electronic applications, which would further promote the development of perovskite devices.

## 2 Results and Discussion

### 2.1 Achieving (111) and (001)‐Oriented Perovskite Film By Antisolvents Engineering

**Figure 1:**
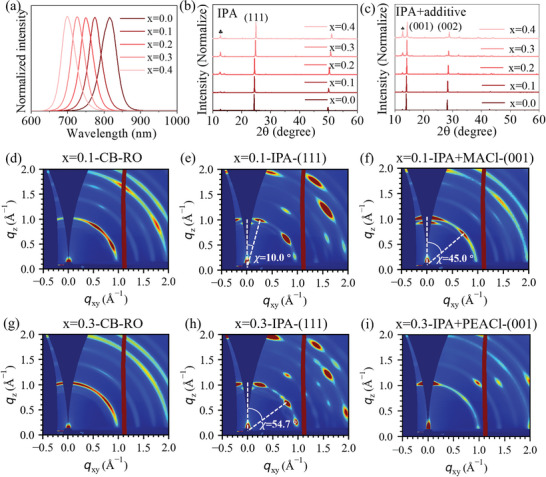
a) Photoluminescence (PL) of perovskite films with varying compositions: Cs_0.03_(FA_0.90_MA_0.10_)_0.97_Pb(I_1‐x_Br_x_)_3_, where x = 0.1, 0.1, 0.2, 0.3, 0.4. b,c). XRD results of perovskite films treated by b) IPA, c) IPA + MACl/PEACl. d–f) GIWAXS patterns obtained from perovskite films with x = 0.1 treated by d) CB, e) IPA, and f) IPA + MACl. g–i) GIWAXS patterns obtained from perovskite films with x = 0.3 treated by g) CB, h) IPA, and i) IPA + PEACl.

The (111)‐ and (001)‐oriented perovskite films were obtained by antisolvent engineering. Cs_0.03_(FA_0.90_MA_0.10_)_0.97_Pb(I_1‐x_Br_x_)_3_ (x = 0‐0.4) perovskite films with the bandgap ranging from 1.53 to 1.77 eV were fabricated by one‐step solution process (**Figure** **1**a; Figure S1 and Table S1, Supporting Information). The details of the fabrication process are shown in the Experimental Section. As we previously reported, high‐quality perovskite films with (111) preferred orientation can be achieved by using a short‐chain isomeric alcohol antisolvent of isopropanol (IPA).^[^ ^31^ ^]^ In this work, the (001) oriented films were achieved by adding specific additives into the IPA antisolvent. The effect of antisolvent on the orientation of perovskite films with different bandgaps was investigated by X‐ray diffraction (XRD) and grazing‐incidence wide‐angle X‐ray scattering (GIWAXS). Figure S2a (Supporting Information) shows the XRD of perovskite films treated by IPA with various additives, where methylammonium chloride (MACl) is the most effective additive to induce purer (001) orientation. The XRD peaks at ≈14.04°, ≈19.94°, ≈24.52°, ≈28.3°, and ≈49.8° correspond to the (001), (011), (111), (002), and (222) crystal planes, respectively. The XRD peak marked with “♣”at 12.7° comes from PbI_2_. As shown in Figure 1b and Figure S2b,c (Supporting Information), the perovskite films treated with chlorobenzene (CB) show random orientation (Figure S2b, Supporting Information), while the films treated by IPA and IPA + MACl show (111) (Figure 1b) and (001) (Figure S2c, Supporting Information) orientations, respectively. Notably, the antisolvent engineering works well for a wide range of Br compositions between x = 0.0 to x = 0.4 by properly selecting the antisolvent additives. For the (001)‐oriented films, IPA + MACl antisolvent works well for systems with x≤0.2, while 2‐Phenylethylaminehydrochloride (PEACl) + IPA generates the best (001) orientation for x>0.2 (Figure 1c; Figure S2d, Supporting Information).

The GIWAXS was conducted to characterize the orientations in both in‐plane and out‐of‐plane directions of NBG (x = 0.1) (Figure 1d–f) and WBG (x = 0.3) (Figure 1g–i) perovskite films. The diffraction rings at q_z_ = 1.4 Å^−1^ are assigned to the (111) planes. The rings at q = 1.0 and 2.0 Å^−1^ are assigned the (001) and (002) planes, respectively. The azimuth angle (𝜒) of 10° and 54.7° in Figure 1e,h indicate that the (001) and (002) planes are inclined at 10° and 54.7° with respect to the surface, which agrees with (111)‐oriented films. Similarly, the 𝜒 of 0.0° and 45.0° fit well with the (001)‐dominated perovskite films. These results are consistent with the XRD spectra. The lower signal of (111) plane compared with (001) plane is due to its weaker interaction with the grazing‐incidence X‐ray and the intensity loss of q_z_.^[^ ^44^ ^]^ Compared with CB‐treated film, the IPA and IPA + MACl/PEACl treated films show more focused diffraction spots, indicating the high‐quality perovskite films with well‐aligned orientations of grains. This is a clear demonstration that antisolvent engineering is an effective method to control the crystal orientation of perovskite films.

### 2.2 Properties of (111) and (100)‐Oriented Perovskite Film

**Figure 2:**
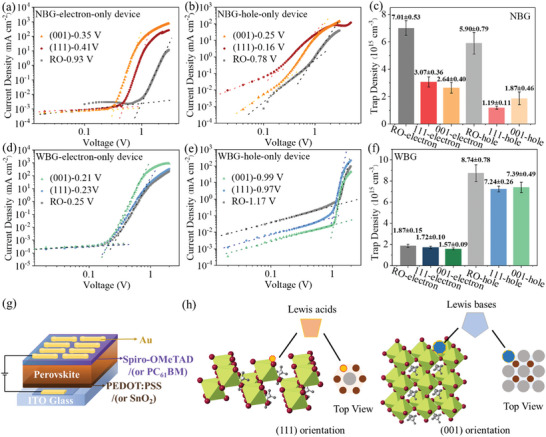
a,b) Average SCLC results of NBG perovskite films treated with different antisolvents based on electron‐only and hole‐only devices. c) Histograms of trap densities of NBG perovskite films based on electron‐only and hole‐only devices. d,e) Average SCLC results of WBG perovskite films treated with different antisolvents based on electron‐only and hole‐only devices. f) Histograms of trap density of WBG perovskite films based on electron‐only and hole‐only devices. g) Diagram of the structure of SCLC devices. h) Diagram of types of defect and corresponding interface passivation agents for (111)‐ and (001)‐oriented perovskite films.

To further investigate the impact of orientation on the properties of perovskite films, space charge limited current (SCLC) was conducted for NBG (x = 0.1, 1.6 eV) and WBG (x = 0.3, 1.7 eV) films. Some targeted precursor additives were added for the defect‐passivation of films with different orientations. The details of the fabrication process are shown in the Experimental Section. Figure S3 (Supporting Information) shows that the precursor additives have little effect on the orientation of perovskite films. Considering the different atomic arrangements on different crystal facets, the defect distribution could differ with the film orientation. The trap density (n_trap_) and carrier mobility (µ) were calculated from SCLC measurements for RO, (111)‐ and (001)‐oriented films in **Figure** **2**a–f, the average trap‐filled limited voltage (V_TFL_) of RO, (111)‐ and (001)‐oriented in NBG perovskite films are 0.93, 0.41, and 0.35 V for the electron‐only device, corresponding to defect density of 7.01 × 10^15^, 3.07 × 10^15^, and 2.64 × 1015 cm^−3^, respectively. For the hole‐only device, the V_TFL_ of RO, (111)‐ and (001)‐oriented perovskite films are 0.78, 0.16, and 0.25 V, corresponding to defect density of 5.90 × 10^15^, 1.19 × 10^15^ and 1.87 × 10^15^ cm^−3^, respectively. The typical dark current density–voltage (*J–V*) characteristics of NBG and WBG for ten electron‐only devices (glass/Indium tin oxide (ITO)/SnO_2_/perovskite/PCBM/Au) and ten hole‐only devices (glass/ITO/PEDOT:PSS/perovskite/Spiro‐OMeTAD/Au) are summarized in Figures S4 and S5 (Supporting Information), and the diagram of the SCLC devices are shown in Figure 2g. These results indicate that the perovskite films with preferred orientation have a lower density than random orientation (RO) films. Moreover, the hole trap density of (111)‐perovskite films is lower than that of (001)‐perovskite films, while the result of electron trap density is exactly the opposite. The carrier mobilities were derived from the SCLC results. The (111)‐oriented films exhibit higher hole mobility but lower electron mobility than that of (001)‐oriented films. The values of carrier trap density and mobility are summarized in Table S2 (Supporting Information). WBG perovskite films exhibit similar phenomena. We speculate that this is related to the different types of defects on (111) and (001) facets, due to their different atomic terminations. As shown in Figure 2h, Pb‐dangling bonds dominate the (111) crystal plane which tends to capture free electrons, thus leading to a higher density of electronic defects. In contrast, the (001) facets have mainly MA vacancies which tend to capture holes and lead to a high density of hole defects. Surface electrical properties of (111)‐ and (001)‐ oriented films measured by the Kelvin Probe Force Microscopy (KPFM) also confirm this speculation. As shown in Figure S6 (Supporting Information), the surface of (111)‐oriented film exhibits lower work function than that of (001)‐oriented film, since the exposed halogens on (111) facets provide sufficient excess electrons, and the exposed cations on the (001) facet acting as the hole doping agents.

Based on the difference in electron/hole trap density and atomic arrangement on (111) and (001) facets, it can be inferred that the (111)‐oriented perovskite films prefer to interact with the electron‐donating group (Lewis acids), whereas the (001)‐oriented perovskite films could be sensitive to electron withdrawing group (Lewis bases). Targeted surface passivation strategies should be designed for perovskite films with different orientations, which will be studied in further research.

**Figure 3:**
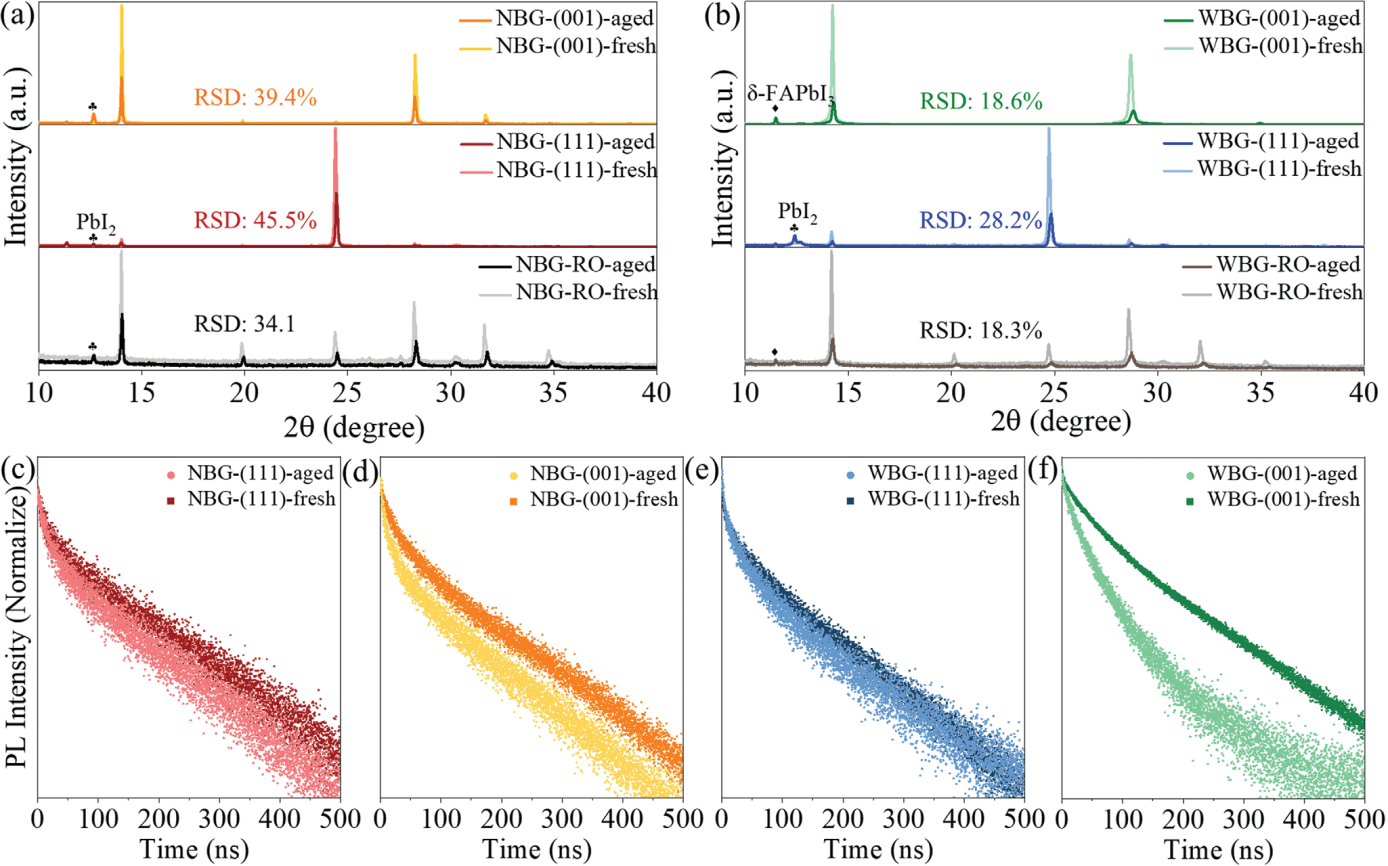
Stability test of different oriented‐perovskite films. XRD and RSD results of fresh and aged samples with RO, (111), and (001) orientation for a) NBG and b) WBG perovskite films. TRPL results of c,d) NBG and e,f) WBG perovskite films with (111) and (001) orientation before and after aging.

Stability tests of NBG and WBG perovskite films with different orientations were tested. The perovskite films were stored in ambient air (16–30 °C, 10–30% relative humidity (RH)) for 30 days and then placed on 85 °C hot stage in air for 72 h for accelerated aging. The XRD spectra of fresh and aged perovskite films are shown in **Figure** **3**a,b. The RO and (111)‐oriented perovskite films exhibit less degradation than the (001) films. The appearance of a peak at ≈12.7° or ≈11.7° indicates the α‐phase perovskite degrades into PbI_2_ or transforms into non‐optical active δ‐FAPbI_3_. Scanning electron microscope (SEM) images of RO, (111), and (001)‐oriented perovskite are consistent with the XRD results (Figure S7, Supporting Information). The relative response spectral degradation ratios^[^ ^45^ ^]^ (RSD: the ratio between the highest XRD peak of the aged film and the fresh film) with different orientations were calculated to quantify the stability of perovskite films. The RSDs of (001)‐ and (111)‐oriented NBG perovskite films are 39.4% and 45.5%, respectively. The RSDs of (001)‐ and (111)‐oriented WBG perovskite films are 18.6% and 28.2%, respectively, proving that (111)‐oriented films have a slight advantage in stability compared to (001)‐oriented films. Time‐Resolved Photoluminescence (TRPL) results before and after aging also confirm this conclusion, as the carrier lifetime of (111)‐oriented films exhibited less deterioration after 2–3 h aging under 85 °C in air (Figure 3c–f; Table S3, Supporting Information).

### 2.3 Orientation‐Dependent Ion Migration

**Figure 4:**
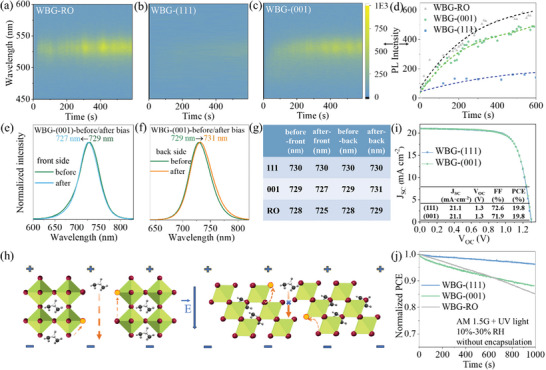
In situ PL of peak position and intensity of a) RO, b) (111)‐oriented, and c) (001)‐oriented WBG perovskite films with laser irradiation time. d) The variation of PL intensity at 530 nm with time of WBG perovskite with different orientations. e,f) PL spectra of the e) front and f) back side of the 001‐oriented film before and after applying bias. g) Statistics of the PL peak positions before and after applying bias. h) Schematic of ion migration in perovskite films under an electric field. i) *J–V* curves and performance of WBG PSCs. j) Stability test of different orientations under the simultaneous irradiation of AM 1.5G and a superimposed UV light (365 nm, 50 mW cm^−2^).

Light‐induced phase separation that significantly limits the PCE and stability of PSCs is a critical issue for I/Br mixed WBG perovskite films. The ion migration under light/electric field has been found to be one of the main reasons for phase separation in PSCs during operation.^[^ ^46^, ^47^, ^48^, ^49^ ^]^ We studied the relationship between film orientation and phase separation in WBG films with different orientations. **Figure** **4**a–c shows the in‐situ PL results of WBG films with RO, (111), and (001) orientations under continuous laser illumination (405 nm, 20 W cm^−2^). All the samples show a new PL peak at ≈ 530 nm (corresponding to the PL peak of Cs_y_(FAMA)_1‐y_PbBr_3_)^[^ ^50^ ^]^ under 405 nm laser irradiation, indicating the phase separation of halide ions and A‐site cations. Figure 4d shows the variation of peak intensity at 530 nm over time. The peak intensity of RO and (001)‐oriented perovskite films increases continuously over time. In contrast, the (111)‐oriented films exhibit much higher phase stability with a very low peak intensity at 530 nm even under prolonged laser irradiation up to 1000 s (Figure S8, Supporting Information).

To further verify the origin of phase separation, devices with structure ITO/perovskite/Au were prepared based on perovskite films with different orientations. A 20 V bias was added with the electric field from Au (front) to ITO (back). The steady‐state PL of the front side and back side of the devices were measured (Figure 4e–g; Figure S9, Supporting Information). Before applying the bias, all films show the same PL peak position on both sides, indicating the homogeneity of perovskite films in the vertical direction. After applying 20 V bias for 5 min, a blue shift of PL peak at the front side and a red shift at the back side was observed for (001)‐oriented and RO films, indicating phase separation caused by the electric field. In contrast, the (111) film shows almost no PL peak shift, exhibiting high resistance to ion migration. These results indicate that the photogenerated electric field is a major cause of ion migration and phase separation in the perovskite films.

The schematics in Figure 4h show the ion migration channels of (001) and (111) films under an external electric field. The (001)‐oriented film provides ion migration channels that are parallel to the electric field, which is most conducive to ion migration and phase separation. This may explain the often‐observed phase separation in WBG perovskites, as most reported WBG perovskite films were (001)‐oriented.^[^ ^51^, ^52^, ^53^ ^]^ In contrast, the tilted lattice of (111)‐oriented film hinders ion migration along the direction of the electric field, which is more suitable for suppressing phase separation of WBG perovskites. One can conclude that the (111)‐oriented film is a good choice for traditional optoelectronic devices such as solar cells, light‐emitting diode, and photodetectors. Although ion migration of the (001)‐oriented film makes it undesirable for solar cells, it provides new opportunities for applications that can utilize ion migration, such as memristors, spectrometers, ionic patterning, and energy storage.^[^ ^54^, ^55^, ^56^ ^]^

The current density–voltage (*J–V*) curves of WBG PSCs are shown in Figure 4i and Figure S10 (Supporting Information). The (111)‐oriented PSCs show a PCE of 19.8%, a open‐circuit voltage (V_OC_) of 1.3 V, a short‐circuit current (J_SC_) of 21.1 mA cm^−2^, and an fill factor (FF) of 72.6%. The (001)‐oriented PSCs show a PCE of 19.8% with a V_OC_ of 1.3 V, a J_SC_ of 21.1 mA cm^−2^, and an FF of 71.9%. We further compared the stability of unencapsulated WBG PSCs by tracking the PCE evolution at maximum power point, as shown in Figure 4j. Three devices for each group were involved to ensure reliability (Figure S11, Supporting Information). To accelerate the phase separation, PSCs were operating under AM 1.5G and a superimposed ultraviolet (UV) illumination (365 nm, 50 mW cm^−2^). The (111)‐oriented PSCs retained ≈97% initial efficiency after 1000 s MPP tracking test, exhibiting the best working stability under intense illumination. However, the efficiencies of (001)‐oriented and RO PSCs show severe degradation as the PCE quickly drops to 88% and 85% of the initial efficiency, respectively. The fast degradation could be attributed to the severe ion migration and phase separation of perovskite films under high‐intensity light and heat, as well as new defects caused by the ion migration.

### 2.4 Device Performance

**Figure 5:**
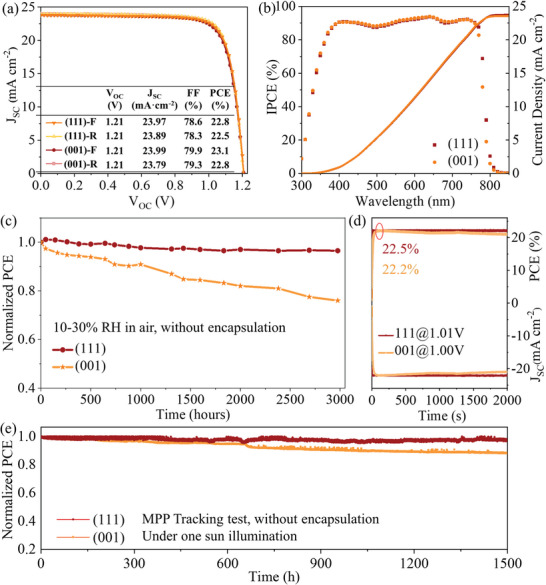
Performance of NBG PSCs based on (111)‐ and (001)‐oriented films. a) *J–V* curves and performance parameters, F and R represent the results of forward and backward scanning tests, respectively. b) IPCE values and its integrated J_SC._ c) Stability test of unencapsulated devices stored in the air. d) SPO results. e) MPP tracking results under one sun illumination.

Solar cells based on (111)‐ and (001)‐oriented perovskite films were fabricated and investigated to reveal the effect of orientation on device performance. We adopt the n‐i‐p structure (ITO/SnO_2_/perovskite/Spiro‐OMeTAD/Au) for PSCs. The typical *J–V* characteristics and photovoltaic parameters of devices based on (111)‐ and (001)‐NBG devices are shown in **Figure** **5**a. The (111)‐oriented perovskite shows a PCE 22.8%, with a V_OC_ of 1.21 V, a J_SC_ of 23.97 mA cm^−2^, and an FF of 78.6% at the forward scan. The (001)‐oriented perovskite device shows a similar PCE of 23.1% with a V_OC_ of 1.21 V, a J_SC_ of 23.99 mA cm^−2^, and an FF of 79.9% at the forward scan. As shown in Figure 5b, the incident photon‐to‐current conversion efficiency (IPCE) values and integrated J_SC_ of champion devices with (111) and (001) orientation are well matched with the *J–V* curves. Figure S12 (Supporting Information) shows the statistics of 50 devices based on (111) and (001) orientations. For long‐term stability, the unencapsulated (111)‐oriented perovskite device retained ≈97% of its original PCE after aging in the air for 3000 h, whereas the (001)‐oriented PSCs only retained ≈76% initial PCE (Figure 5c). The stable power output (SPO) of the (111)‐oriented device was 22.5% at a bias of 1.01 V, whereas the (001)‐oriented device was 22.2% at a bias of 1.00 V (Figure 5d, red circle). After a 30 min test, the (001)‐oriented PSC showed a slight light‐induced degradation and the PCE dropped to 95% of its initial value, which is a common issue in PSCs with a normal device structure. In contrast, the (111)‐oriented PSC exhibited almost no degradation and maintained ≈100% of its initial PCE with a stable photocurrent output (Figure 5d). Furthermore, the operating stability of unencapsulated devices was tested under a nitrogen atmosphere at room temperature. As shown in Figure 5e, the (111)‐oriented PSC remained at 95% of its initial efficiency after 1500 h MPP tracking test under one sun illumination, while the (001)‐oriented PSC slightly dropped to 90% of its initial efficiency. The excellent working stability of (111)‐oriented PSCs could be mainly attributed to the superior environmental and phase stability of (111)‐oriented perovskite films. For (001)‐oriented PSCs, targeted water/oxygen protection and surface modification strategies should be designed to improve the film and device stability.

## 3 Conclusion

We achieved (111)‐ and (001)‐oriented perovskite films with bandgap ranging from 1.53 to 1.77 eV by simple antisolvent engineering with a universal method. This provides an objective way to compare the effects of orientation on the intrinsic properties of perovskite films, eliminating the effects of precursor additives, which were commonly used in previous reports. We therefore performed comprehensive studies on the orientation‐dependent film properties including defect distribution, ion migration, carrier mobility, and photo/electric stability for perovskite films based on RO‐, (111)‐, and (001)‐orientations. We found that the intrinsic film properties were highly dependent on the film orientation. Therefore, targeted defect passivation and application scenarios for different film orientations were necessary and have been discussed to further improve the device's performance. The (111)‐oriented films exhibit higher environmental stability due to their inorganic‐dominated surface termination. Ion migration and phase separation were also studied to address these highly concerned issues in WBG perovskite films. The (111)‐oriented film could effectively suppress the phase separation as the tilted lattice hinders ion migration stimulated by an electric field. In contrast, the (001)‐oriented films exhibit pronounced ion migration, leading to severe phase separation in WBG films, which could be utilized in other electronic applications. As a result, the (111)‐oriented PSCs exhibit higher stability than the (001) groups with a shelf lifetime of T_97_ = 3000 h in an ambient environment. This work paves the way for rational design and controllable synthesis of uniaxial‐oriented perovskite films. The systematic analysis of orientation‐dependent properties provides guidance for the design of novel electronic applications of perovskites, which would further promote the development of perovskite photovoltaic and optoelectronic devices.

## 4 Experimental Section

### Materials

Unless otherwise stated, all chemicals were purchased from Tokyo Chemical Industry Co., Ltd., and Xi'an Polymer Light Technology Crop., and all solvents were purchased from J&K Scientific Ltd. And used as received. The SnO_2_ colloid precursor was purchased from Alfa Aesar (Tin (IV) oxide, 15% in H_2_O colloid dispersion).

### Film Fabrication of Perovskite

1.5 m Cs_0.03_(FA_0.90_MA_0.10_)_0.97_Pb(I_x_Br_1‐x_)_3_ (x = 0.0–0.4) perovskite precursor was obtained by dissolving FAI, MAI, CsI, PbBr_2_, PbI_2_ and 5% mmol RbCl in a mixed solvent of DMF:DMSO = 4:1, and adopted a one‐step spin‐coating process with a two‐step spin‐coating procedure (2000 rpm for 10 s followed by 6000 rpm for 20 s.). For (111)/(001)‐perovskite, IPA/IPA+additive was dropped onto the spinning substrate during the second spin‐coating step at the 12 s. The additive in IPA for NBG is 3 mg mL^−1^ MACl and for WBG is 1 mg mL^−1^ PEACl. For randomly oriented perovskite, CB was dropped onto the spinning substrate during the second spin‐coating step at 12 s. The substrate was then immediately transferred on a hotplate and annealed, 120 °C annealed for 20 min for NBG perovskite film and 140 °C annealed for 20 min for WBG perovskite film.

### Device Fabrication

For perovskite devices, the glass/ITO substrates were sequentially cleaned using distilled water, acetone, and isopropanol. The SnO_2_ electron transporting layer was obtained by dissolving SnO_2_ colloid dispersion in distilled water (SnO_2_: H_2_O = 1:6) and ultrasound for 4 min before spin‐coated (4000 rpm, 30 s) on glass/ITO substrates in ambient air and annealed at 170 °C for 30 min.^[^ ^57^, ^58^ ^]^ Perovskite films were then deposited on the as‐prepared substrate. After cooling down to room temperature, Spiro‐OMeTAD (72.3 mg mL^−1^ in CB), tert‐butylpyridine (29 µL mL^−1^), and bis(trifluoromethane)sulfonimide lithium salt (18.5 µL mL^−1^, 520 mg mL^−1^ in acetonitrile) was subsequently deposited on top of the perovskite film by 4000 rpm for 30 s. Finally, 55 nm of gold electrodes were deposited on top of the devices by thermal evaporation in a vacuum of≈10^−5^ Torr. For the SCLC test, the device structure of the electron‐only device was glass/ITO/SnO_2_/perovskite/PCBM/Au and the structure of hole‐only device was glass/ITO/PEDOT: PSS/perovskite/Spiro‐MeOTAD/Au. All layers were fabricated using the same method as the solar cells except PEDOT: PSS and PCBM. The PEDOT: PSS layer was spin‐coated (4000 rpm 30 s) on the ITO/glass substrates in ambient air, and then annealed at 135 °C for 10 min. The PCBM layer was obtained by dissolving PC_61_BM in CB (10 mg mL^−1^) and spinning‐coated at 2000 rpm for 30 s.

### Device and Film Characterization

The current–voltage (*J–V*) characteristics of devices were tested via a Keithley 2635B source meter under simulated AM 1.5G (100 mW cm^−2^) solar irradiation in the air. A calibrated silicon solar cell from Newport Corp. was used to calibrate the light intensity. The *J–V* curves were tested from 1.3 to −0.1 and −0.1 to 1.3 V with a scanning speed of 0.02 V s^−1^. The masked active area was 0.0805 cm^2^. The surface morphology of the perovskite films was characterized by a FEI Apreo C with an electron beam energy of 10 kV. TRPL spectra were measured on a platform built by ourself base on RedWave Labs APD using a 405 nm pulse laser and the light was illuminated from the perovskite film side. The KPFM was performed on perovskite samples in the air using AFM (Bruker Dimension Icon). For KPFM, the scanning was done in lift mode, during the first scanning the surface topography was directly obtained, and second scanning gave the surface potential information. The PL was executed by A laser scanning confocal microscope (Self‐built platform) equipped with a 405 pulse laser and Mach‐DSP scanner. XRD patterns were recorded on a Rigaku smartlab X‐ray Diffractometer. The GIWAXS data were obtained at 1W1A Diffuse X‐ray Scattering Station, Beijing Synchrotron Radiation Facility (BSRF‐1W1A). EQEs were measured by a solar cell quantum efficiency measurement system (QE‐R) supported by Enli Technology Co., Ltd. The absorption spectra were measured by a Hitachi UH4150 spectrophotometer. All the tests were taken in the ambient air at a temperature of 18–35 °C and relative humidity ≈ 10–30% except for SEM.

### Statistical Analysis

All statistical analysis was performed with OriginPro 2021. The original data obtained from PL, TRPL, and XRD in Figure 1, Figure S2a,b (Supporting Information) were normalized. The other data were obtained by transferring the corresponding original data according to the calculation formula. Linear fitting was applied to SCLC. The biexponential decay function was applied to TRPL decays to infer the carrier extraction/recombination dynamics. GIWAXS (Jianyao Huang, GIWAXS Tools, Version [current version], https://gitee.com/swordshinehjy/giwaxs script (accessed date).

## Conflict of Interest

The authors declare no conflict of interest.

## Supporting information

Supporting Information

## Data Availability

The data that support the findings of this study are available from the corresponding author upon reasonable request.
