# Supplementary material for: Uniaxial‐Oriented Perovskite Films with Controllable Orientation
Source: Adv Sci (Weinh). 2024 Mar 11;11(19):2401184. doi: 10.1002/advs.202401184 (PMC11109632; doi:10.1002/advs.202401184)
Supplement: Supplementary file 1 — Supporting Information [file ADVS-11-2401184-s001.pdf]

ADVANCED SCIENCE
Open Access

Supporting Information

for *Adv. Sci.*, DOI 10.1002/advs.202401184

Uniaxial-Oriented Perovskite Films with Controllable Orientation

Dongni Li, Xiangyu Sun, Yao Zhang, Zhen Guan, Yansong Yue, Qingya Wang, Lu Zhao, Fangze Liu, Jing Wei and Hongbo Li**

((Supporting Information can be included here using this template))

Supporting Information

Uniaxial-Oriented Perovskite Films with Controllable Orientation

Dongni Li, Xiangyu Sun, Yao Zhang, Zhen Guan, Yansong Yue, Qingya Wang, Lu Zhao, Fangze Liu, Jing Wei and Hongbo Li**

D. Li, X. Sun, Y. Zhang, Z, Guan, Y. Yue, Q. Wang, L. Zhao, F. Liu, J. Wei, H. Li

Beijing Key Laboratory of Construction-Tailorable Advanced Functional Materials and Green

Applications

Experimental Center of Advanced Materials

School of Materials Science and Engineering

Beijing Institute of Technology

Beijing 100081, China

E-mail: weijing@bit.edu.cn; hongbo.li@bit.edu.cn

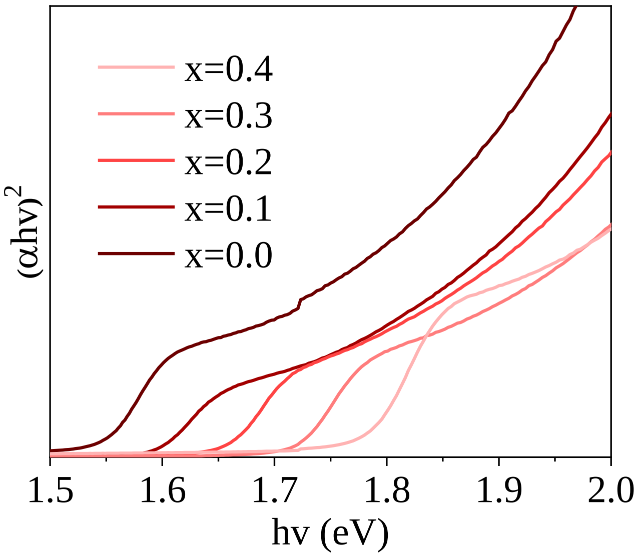

Figure S1. UV-visible absorption spectra of perovskite films with varying compositions: $Cs_{0.03}(FA_{0.90}MA_{0.10})_{0.97}Pb(I_{1-x}Br_x)_3$, where x=0.1, 0.1, 0.2, 0.3, 0.4.

Table S1: The bandgap of varying $Cs_{0.03}(FA_{0.90}MA_{0.10})_{0.97}Pb(I_{1-x}Br_x)_3$ (x=0.0-0.4) compositions

x	0	0.1	0.2	0.3	0.4
PL	1.53	1.6	1.65	1.70	1.77
UV-Vis	1.55	1.59	1.66	1.72	1.78

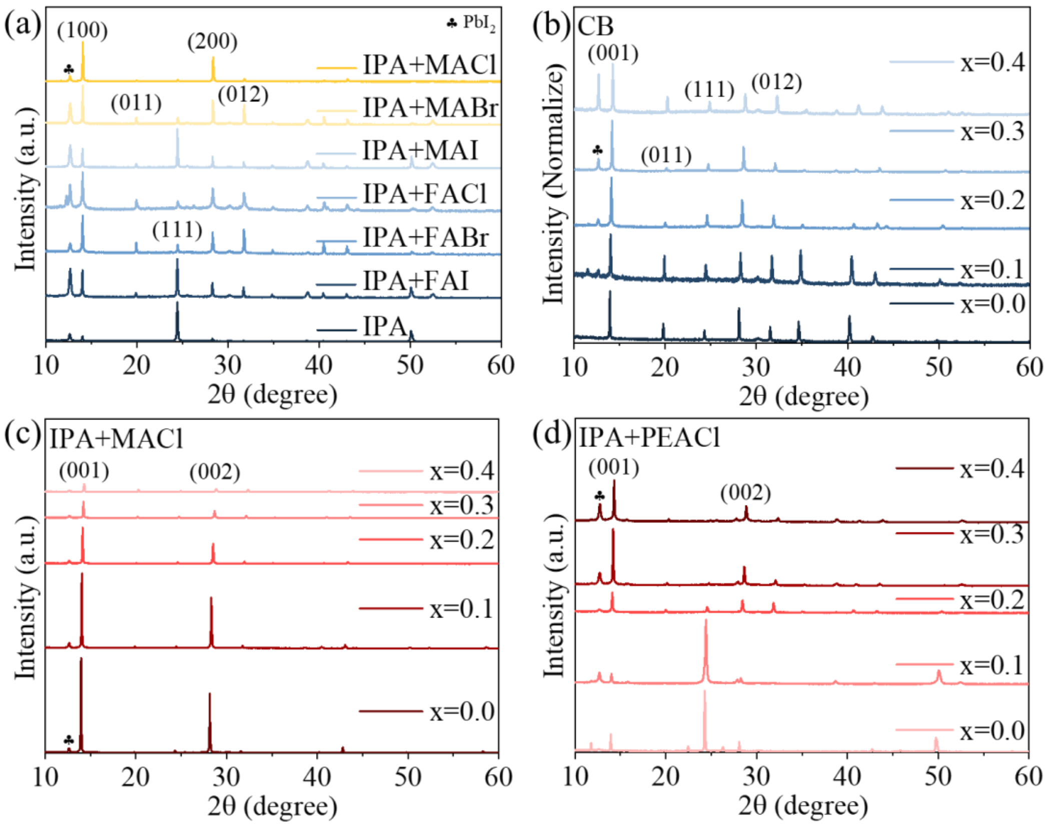

Figure S2. XRD of perovskite films prepared with a) different IPA + additives, b) CB, c) IPA + MACl and d) IPA + PEACl.

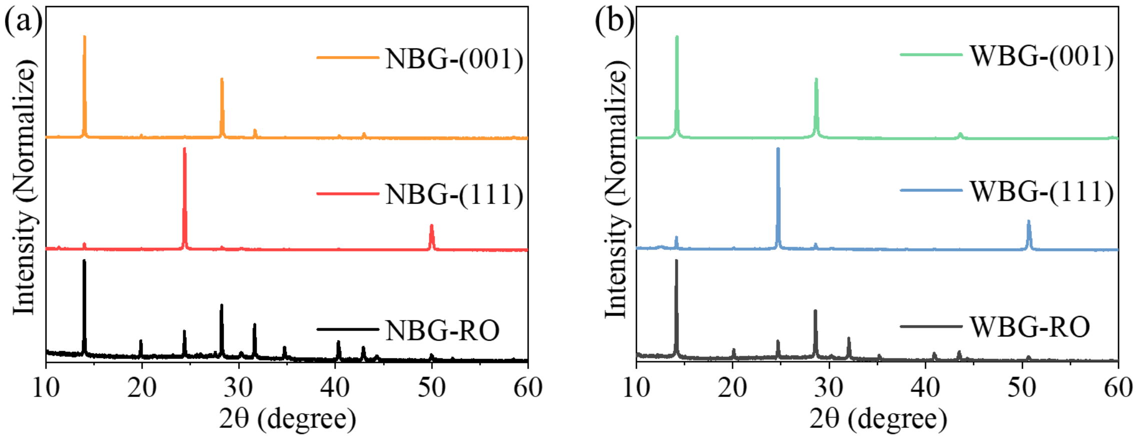

Figure S3. XRD of a) NBG and b) WBG with precursor additives.

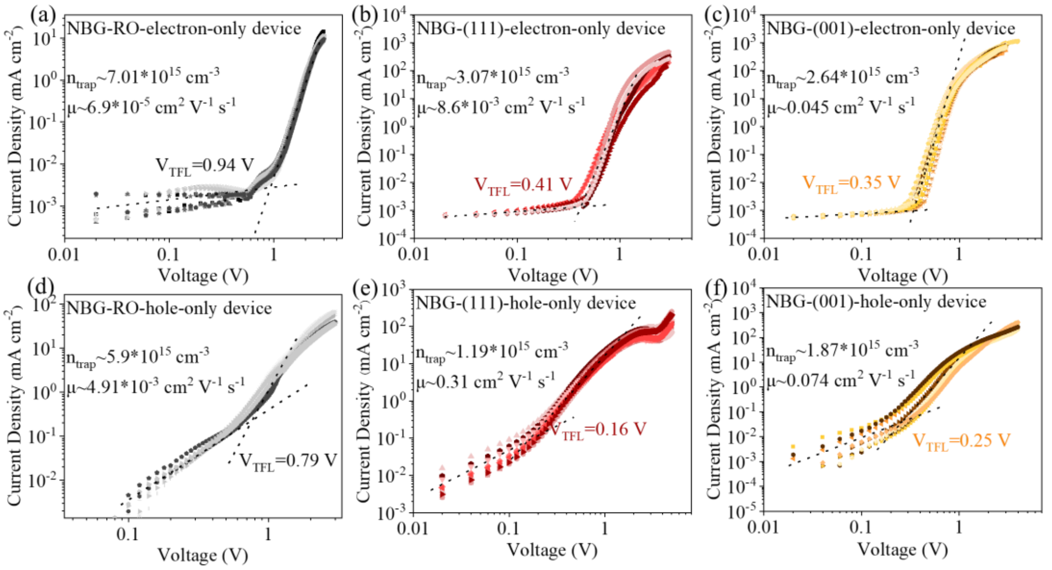

Figure S4. SCLC results of NBG films treated with a, d) CB, b, e) IPA and (c, f) IPA+MACl. The dark -current-voltage curves of a), b), c) based on electron-only device and d), e), f) based on hole-only device.

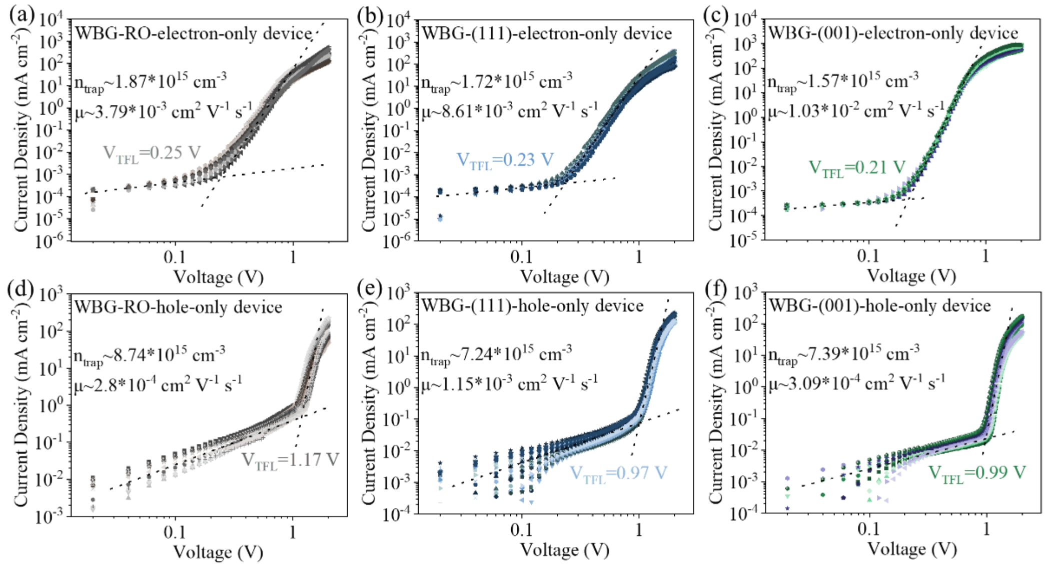

Figure S5. SCLC results of WBG films treated with a, d) CB, b, e) IPA and (c, f) IPA+PEACl. The dark -current-voltage curves of a), b), c) based on electron-only device and d), e), f) based on hole-only device.

Table S2. The values of carrier trap density and mobility for NBG and WBG perovskite with different orientation. The results were average value extracted from 10 samples for each group.

	Electron-n_{trp} (cm^{-3})	Electron-μ ($cm^2V^{-1}s^{-1}$)	Hole-n_{trap} (cm^{-3})	Hole-μ ($cm^2V^{-1}s^{-1}$)
NBG-RO	$7.01*10^{15}$	$6.9*10^{-5}$	$5.9*10^{15}$	$4.91*10^{-3}$
NBG-(111)	$3.07*10^{15}$	$8.6*10^{-3}$	$1.19*10^{15}$	$3.1*10^{-1}$
NBG-(001)	$2.64*10^{15}$	$4.5*10^{-2}$	$1.87*10^{15}$	$7.4*10^{-2}$
WBG-RO	$1.87*10^{15}$	$3.79*10^{-3}$	$8.74*10^{15}$	$2.8*10^{-4}$
NBG-(111)	$1.72*10^{15}$	$8.61*10^{-3}$	$7.24*10^{15}$	$1.15*10^{-3}$
WBG-(001)	$1.57*10^{15}$	$1.03*10^{-2}$	$7.39*10^{15}$	$3.09*10^{-4}$

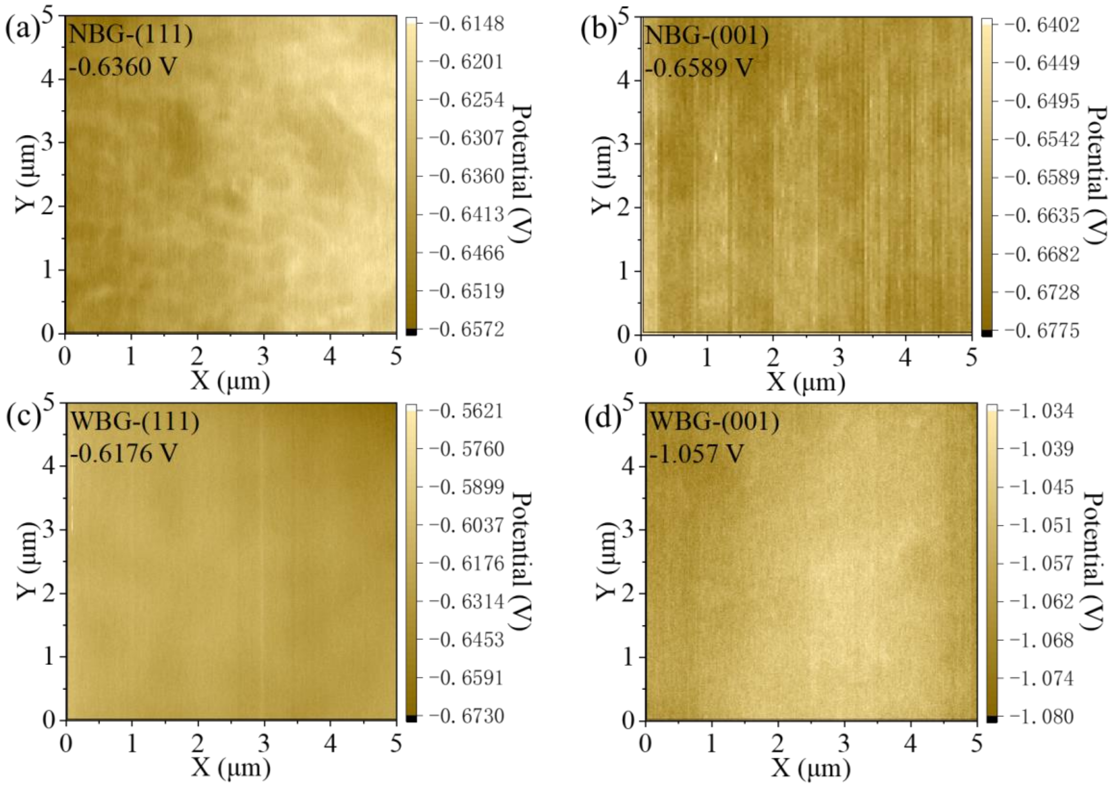

Figure S6. KPFM of NBG perovskite films treated with a) (111) and b) (001) orientation. KPFM of WBG perovskite films treated with c) (111) and d) (001) orientation. The number in the Figure indicate the average potential of each film.

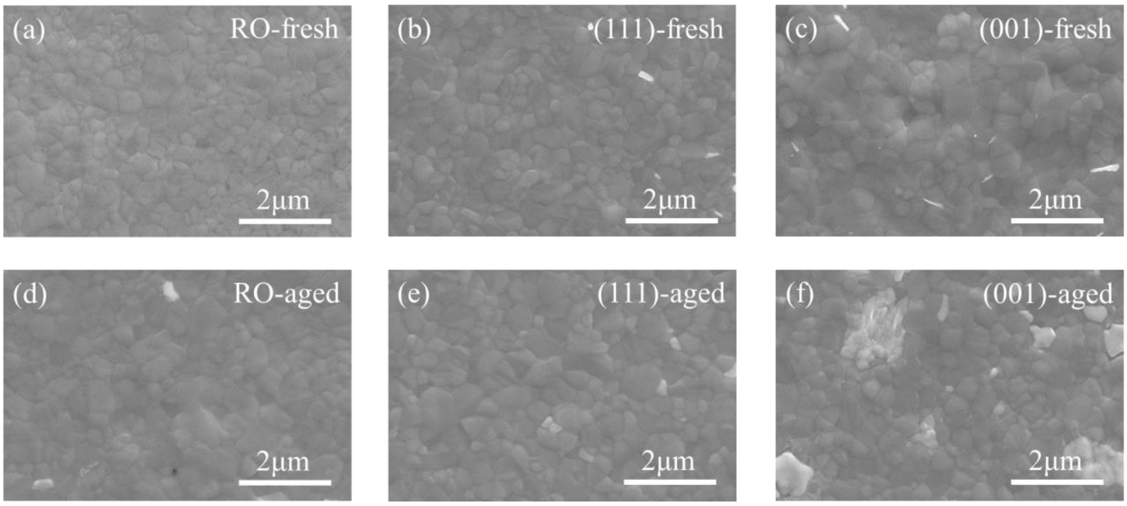

Figure S7. SEM images of NBG perovskite film using a) CB, b) IPA and c) IPA+MACl as antisolvent before and after 85 °C thermal aging in air.

Table S3. TRPL parameters of NBG and WBG films with (111) and (001) orientation before and after aging.

	τ_1 (ns)	τ_2 (ns)	τ_{aver} (ns)
NBG-(111)-fresh	25.72	830.95	811.25
NBG-(111)-aged	23.49	646.47	617.53
NBG-(001)-fresh	42.41	813.29	791.85
NBG-(001)-aged	20.48	516.38	480.15
WBG-(111)-fresh	23.36	326.44	294.96
WBG-(111)-aged	19.62	248.2	200.05
WBG-(001)-fresh	46.18	253.92	222.36
WBG-(001)-aged	31.00	122.27	87.61

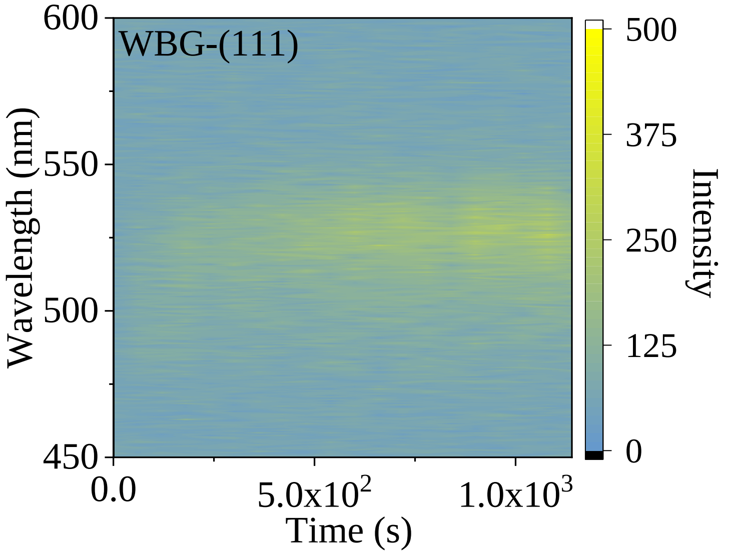

Figure S8. PL of peak position and intensity changes with laser irradiation time of WBG perovskite film using IPA as antisolvent.

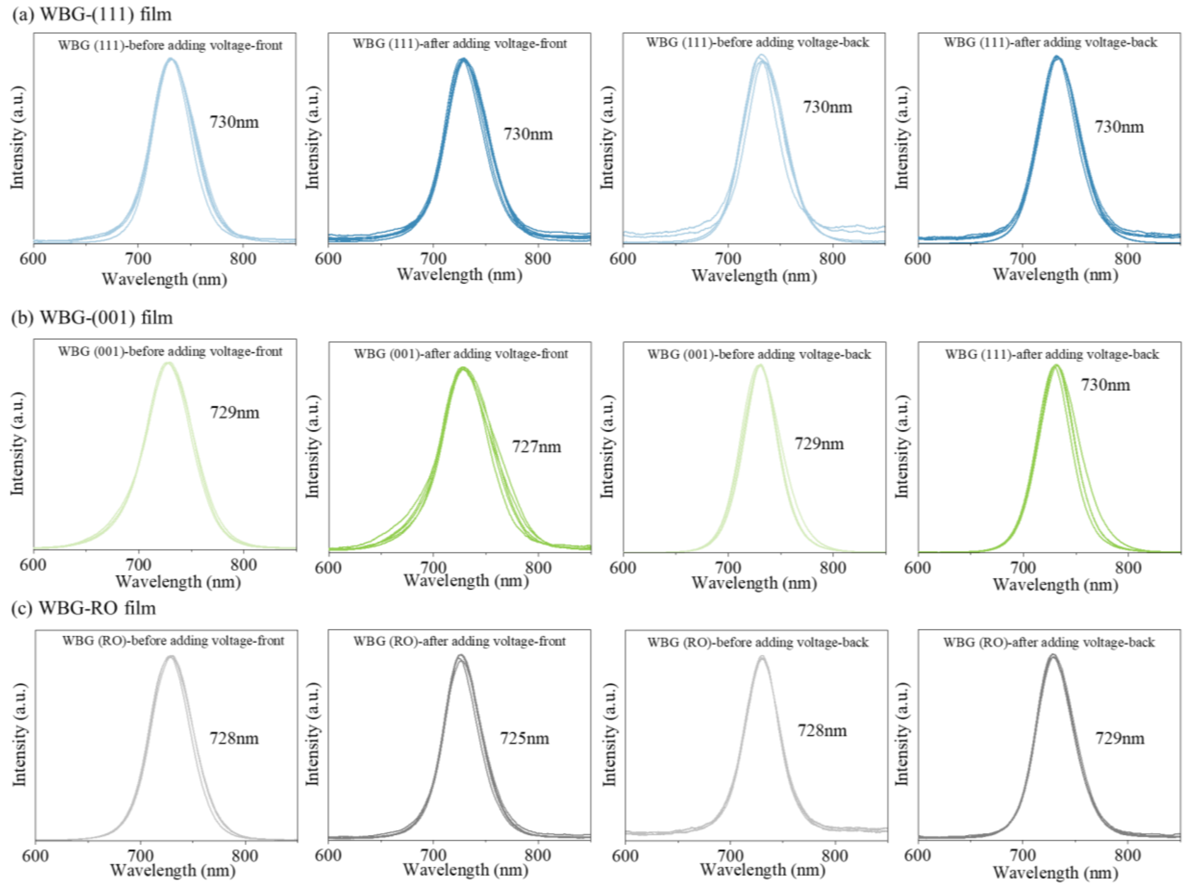

Figure S9. PL spectra of the WBG films on the front and back sides before and after applying bias voltage with a structure of Au/perovskite/ITO.

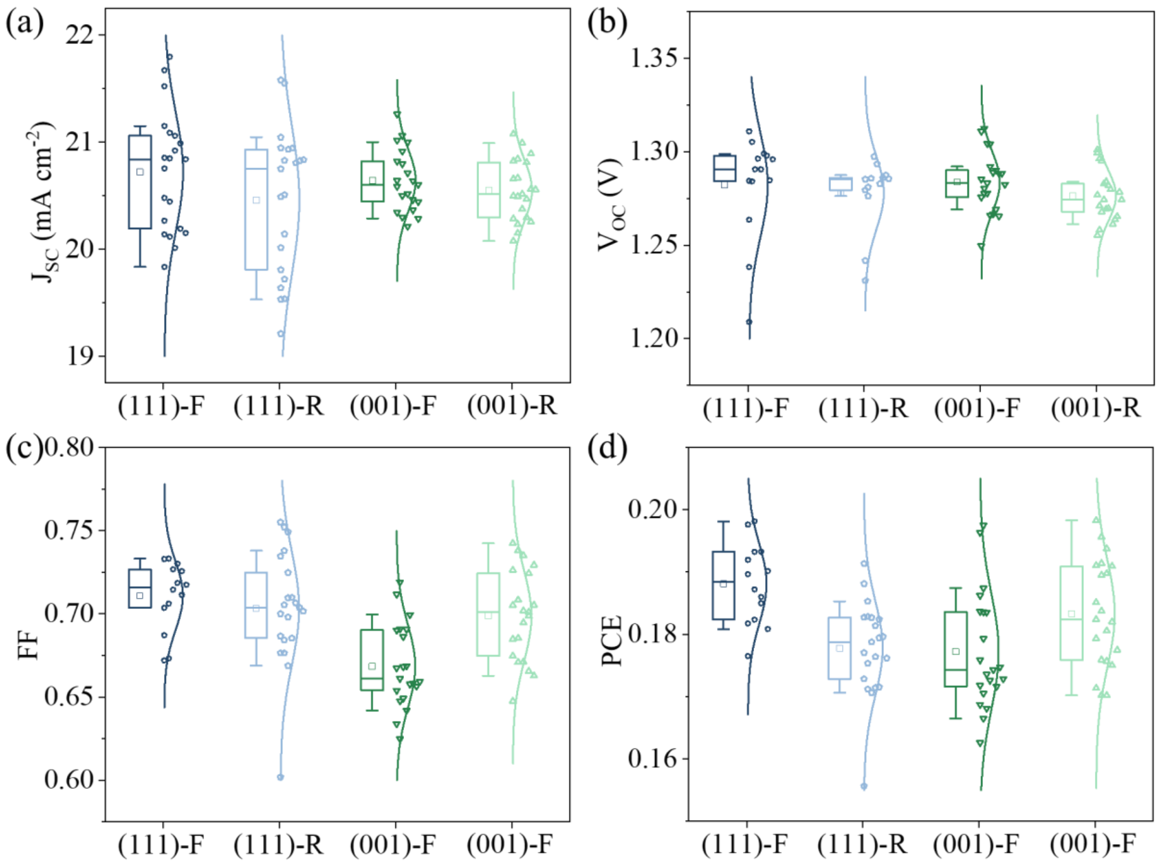

Figure S10. Histograms of the a) J_{SC}, b) V_{OC}, c) FF and d) PCE for the (111)- and (001)-oriented WBG devices.

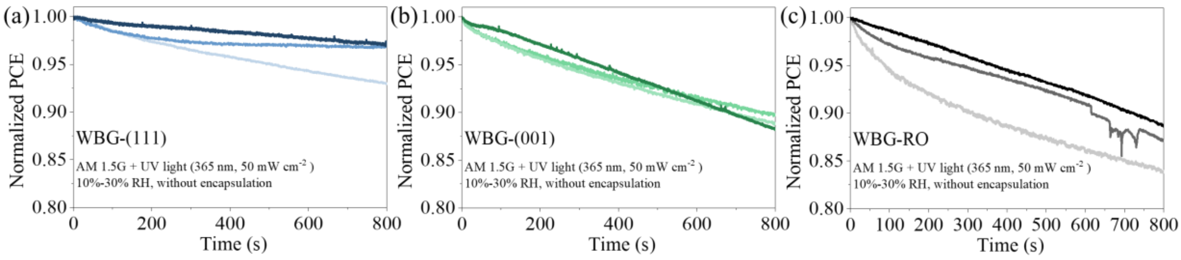

Figure S11. Stability test of different orientation under the simultaneous irradiation of AM1.5G and a superimposed UV light (365 nm, 50 mW/cm^2).

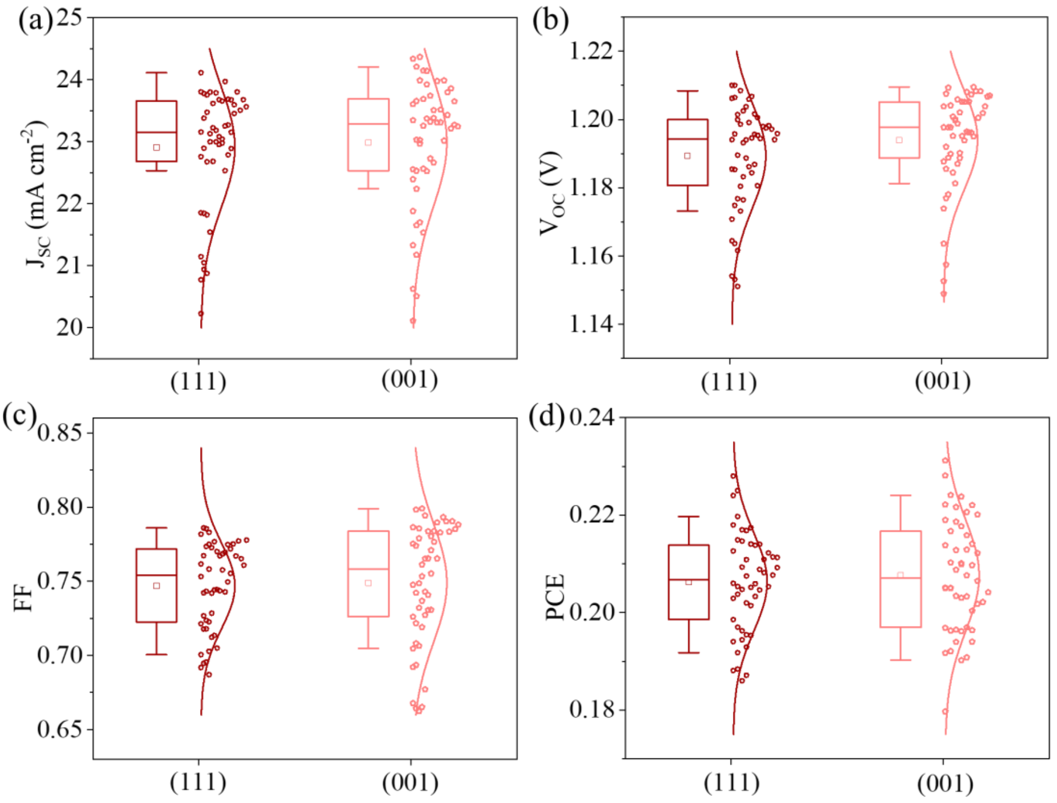

Figure S12. Histograms of the a) J_{SC}, b) V_{OC}, c) FF and d) PCE for the (111)- and (001)-oriented NBG-PSCs.
